# Ultrasound measurements versus invasive intracranial pressure measurement method in patients with brain injury: a retrospective study

**DOI:** 10.1186/s12880-019-0354-0

**Published:** 2019-07-05

**Authors:** Jing Zhou, Jing Li, Tiantian Ye, Yanrong Zeng

**Affiliations:** Department of Ultrasound, Beijing Luhe Hospital, Affiliated to Capital Medical University, Beijing, 101149 China

**Keywords:** Blood flow velocities, Brain injury, Intracranial pressure, Optic nerve sheath diameter, Pulsatility index, Ultrasound

## Abstract

**Background:**

The invasive method for intracranial pressure measurement is ‘gold standard’ but not always feasible because the intraventricular catheter/ intraparenchymal micro transducer used in the measurement of intracranial pressure measurement may cause complications. Imaging modalities with clinical examination protocol have a lack of specificity and accuracy. The objective of the study was to compare the accuracy of diagnostic parameters of ultrasound measurements in patients with brain injury underwent invasive intracranial pressure measurement method.

**Methods:**

Data of invasive intracranial pressure measurement method and ultrasound measurements of 185 patients with brain injury who required admission diagnosis were included in the analysis. Pearson correlation was tested for diagnostic parameters. Logistical regression analysis was performed for diagnostic parameters of death patients to evaluate independent parameter of mortality.

**Results:**

Straight sinus flow velocities, middle cerebral artery flow velocities, and optic nerve sheath diameter were correlated with intracranial pressure (*p* < 0.0001 for all). Arterial blood pressure (*p* = 0.127) and middle cerebral artery pulsatility index (*p* = 0.06) were not correlated with intracranial pressure. A total of 47 patients died during the study period. Intracranial pressure (*p* = 0.015) and optic nerve sheath diameter (*p* = 0.035) were found to be independent predictor of mortality.

**Conclusions:**

Ultrasound measurement especially optic nerve sheath diameter can be successfully used instead of invasive intracranial pressure measurement method in patients with brain injury.

**Level of evidence:**

III.

## Background

Intracranial hypertension is a deadly complication and one of the contributing factor in secondary brain injury because its intensity and duration are associated with a fatal outcome [1].

Imaging modalities with clinical examination protocol have a lack of specificity [2] and insufficient accuracy [3]. Therefore, the invasive intracranial pressure measurement method is recommended [4] but a multicenter, controlled trial on patients with brain injury comparing care focused on imaging and clinical examination with care focused on maintaining intracranial pressure at 20 mmHg or less has found no significant differences [2].

The use of an intraventricular catheter/ intraparenchymal micro transducer may cause complications like infection or hemorrhage [5]. Therefore, the invasive intracranial pressure measurement method is only used in severe traumatic brain injury when systemic complications are absent [6]. When invasive intracranial pressure measurement method is contraindicated, imaging modalities with clinical examination would be preferred [4]. A prospective, observational study on patients with traumatic brain injuries recommended ocular sonography as a screening test for raised intracranial pressure measurements [7]. A retrospective study suggested transcranial Doppler for measurement of intracranial pressure [8]. Two-depth transcranial Doppler has better diagnostic reliability for intracranial pressure measurements [9, 10].

The primary aim of the retrospective study was to compare the accuracy of diagnostic parameters of ultrasound measurements in patients with brain injury underwent invasive intracranial pressure measurement method. The secondary endpoint of the study was to evaluate independent diagnostic parameters for mortality of patients in a Chinese setting.

## Methods

### Inclusion criteria

Patients age 18 years or older with severe traumatic brain injury, intraparenchymal hemorrhage, aneurysmal subarachnoid hemorrhage, or stroke (decision of neurosurgeon) were included in the analysis. Among these patients, who required sedation, intracranial pressure monitoring or mechanical ventilation with an admission diagnosis were included in the analysis.

### Exclusion criteria

Patients were excluded from the analysis who had optic nerve injuries, skull base fracture (with cerebrospinal fluid leaks), an ocular pathology, inaccessible ultrasound windows, and no radiological or clinical susceptibility of cerebral venous vasospasm or thrombosis. Patients whose intracranial pressure mean values changed > 2 mmHg during ultrasound measurements and who had a history of glaucoma were not included in the analysis.

### Clinical management of patients

Patients were put on propofol (a continuous infusion) and the muscle relaxant (atracurium) was given when necessary. Mechanical ventilation was adjusted to maintain normocapnia (partial pressure of carbon dioxide: 38 ± 2 mmHg) and adequate oxygenation (peripheral oxygen saturation ≥ 91%). The adequate cerebral perfusion pressure had been maintained ≥61 mmHg by norepinephrine, vasopressors, and normal saline [11]. As per institutional guidelines, intracranial hypertension had been treated by optimizing arterial blood pressure and using hyperosmolar fluids and sedation infusion.

### Invasive intracranial pressure measurement method

Intracranial pressure had been measured by intensive care physician (minimum 10-years of experience) of institutes using 1.3 mm Tunnelling intraparenchymal probe (3PN, Spiegelberg GmbH & Co. KG, Hamburg, Germany) or a catheter inserted into the ventricles of brain and was connected to an external drainage and pressure transducer and system (Camino®, Integra® Life Sciences Corporation, Plainsboro, NJ, USA). Admission Glasgow Coma Scale, demographical characteristics, comorbidities, severity and mechanism of brain injuries, and discharge Glasgow Outcome Scale were recorded for each patient [12].

### Ultrasound measurements

Ultrasound measurements had been performed by ultrasound equipment (GE Healthcare, Beijing, China) by ultra-sonographers of the institutes (blinded regarding the results of the invasive intracranial pressure measurement method and minimum 10-years of experience). The mean values of five individual measurements were considered in the analysis. Venous transcranial Doppler measurements, optic nerve sheath diameter, and arterial transcranial doppler had been measured twice in a day from day 1–5 at 50–80 mm optic nerve blood pressure (as accessible ultrasound windows).

### Venous transcranial Doppler measurements

Arterial blood pressure, cerebral perfusion pressure, the partial pressure of carbon dioxide, diastolic (FV_d_), mean (FV_m_), and systolic (FV_s_) middle cerebral artery flow velocities, FV_d_, FV_m_, and FV_s_ straight sinus flow velocities were recorded by 2 MHz linear probes (GE Healthcare, Beijing, China) on the straight sinus through an occipital and transforaminal bone window [5].

### Optic nerve sheath diameter measurements

**Fig. 1:**
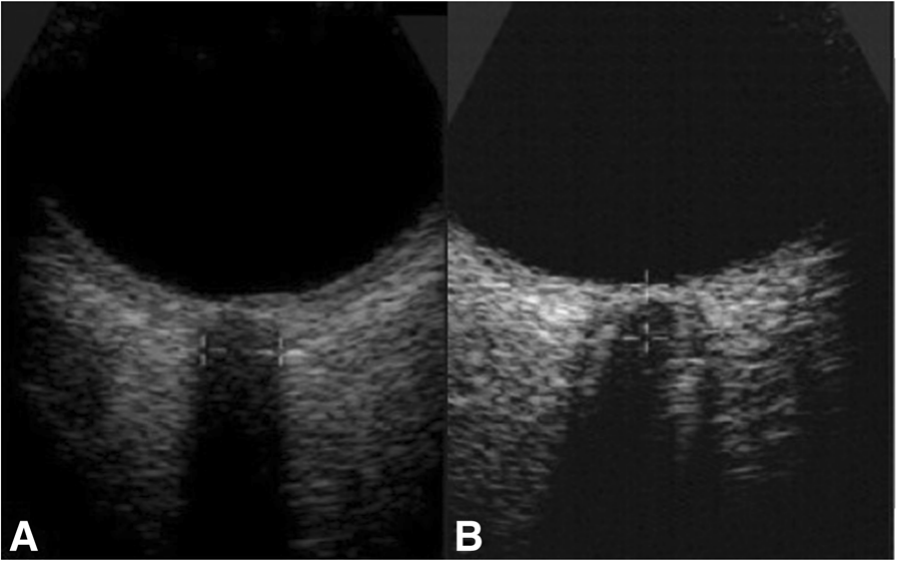
Ultrasound measurements of the axial plane of the right eye. **a** Optic nerve sheath of a patient with normal intracranial pressure. No subarachnoid space around the optic nerve. **b** Optic nerve sheath of a patient with high intracranial pressure. Significant subarachnoid space around the optic nerve

Optic nerve sheath diameter was recorded by 7.5 MHz linear probes (GE Healthcare, Beijing, China) with the lowest possible acoustic power. The gel was applied on the surface of eyelids. The probe was oriented 30^0^ on both closed eyes of the patients with the head elevated to 30^0^ in the supine position at approximately 30^0^ on the length of the plane and at the horizontal surface. The measurements of axial and sagittal planes of both eyes had been made such that the visible widest diameter behind the retina was 2.8 mm (Fig. 1) [13].

### Arterial transcranial Doppler measurement

2 MHz linear probe (GE Healthcare, Beijing, China) was used to measure arterial transcranial Doppler measurement in the same supine position of the patients. It was performed bilaterally on middle cerebral artery using temporal window [14].

Events of mortality were collected from institutional records.

### Statistical analysis

InStat GraphPad Software, San Diego, IL, USA was used for statistical analysis. Pearson correlation was tested for diagnostic parameters. Logistical regression analysis was performed for diagnostic parameters among the data of death patients. Inter-rater reliability was evaluated by intraclass correlation coefficient (> 0.8: optimal, 0.7–0.8: strong, 0.5–0.69: moderate, 0.3–0.49: fair, < 0.3: poor), calculated by a two-way random effects model in Stata 12.1 software (StataCorp, College Station, TX, USA) [15]. The results were considered significant at 95% of confidence level.

## Results

### Enrollment

**Fig. 2:**
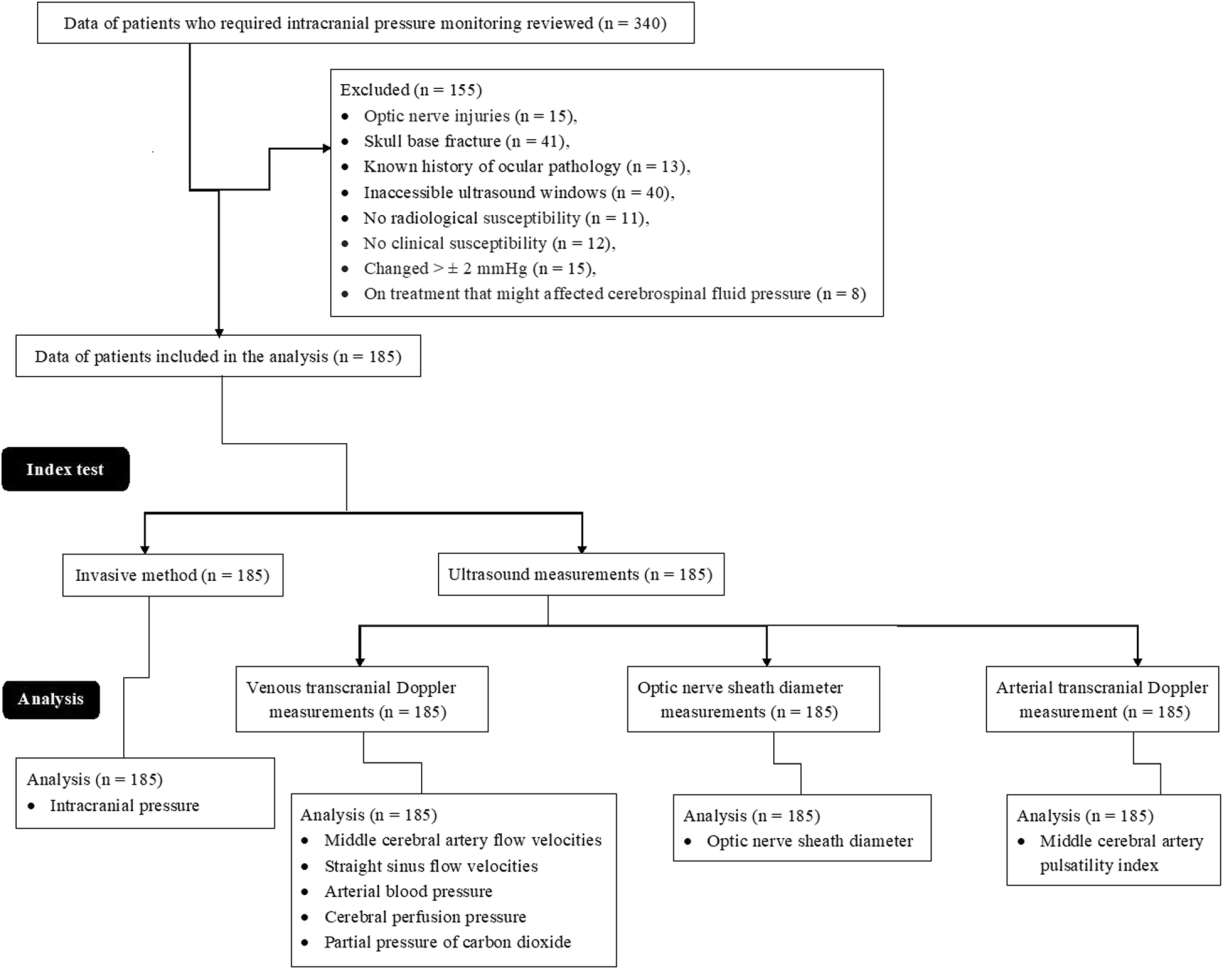
Flowchart of the analysis

Data of patients who required intracranial pressure monitoring or mechanical ventilation with an admission diagnosis from 12 February 2017 to 1 January 2019 of the Beijing Luhe Hospital, Affiliated to Capital Medical University, Beijing, China, and referring hospitals were reviewed. Among the available patients’ records, 15 patients had optic nerve injuries, 41 had skull base fracture, 13 had a known history of ocular pathology, 40 had inaccessible ultrasound windows, 11 had no radiological susceptibility of cerebral venous thrombosis, and 12 had no clinical susceptibility of cerebral thrombosis. Therefore, these data were excluded from the analysis. Also, 8 patients were on treatment that might affect cerebrospinal fluid pressure, so excluded from the analysis. Even, 15 patients had reported more than ±2 mmHg changes in mean values of intracranial pressure during ultrasound measurements. Therefore, these data were also excluded from the analysis. Data of invasive intracranial pressure measurement method and ultrasound measurements of 185 patients were included in the analysis. Flow chart of the study is presented in Fig. 2.

### Characteristics of patients

The minimum admission Glasgow Coma Scale of the enrolled patients was 4. The other demographical and clinical characteristics of enrolled patients are presented in Table 1.

**Table 1:** Demographical and clinical characteristics of enrolled patients

Characteristics		Value
Data of patients included in the analysis		185
Gender	Male	86 (47)
	Female	99 (53)
Age (years)	Minimum	38
	Maximum	68
	Mean ± SD	52.42 ± 4.53
Weight (kg)		58.42 ± 4.59
Height (cm)		161 ± 6
Pathology		
Traumatic brain injury		135 (73)
Intraparenchymal hemorrhage		38 (21)
Aneurysmal subarachnoid hemorrhage		12 (6)
Comorbidities		
Asthma		19 (10)
Hypertension		18 (10)
Depression		35 (19)
Alcoholic		8 (4)
Smokers		15 (8)
History of myocardial infarction		3 (2)
Admission Glasgow Coma Scale	Maximum	14
	Minimum	4
	Median	9
Discharge Glasgow Outcome Scale	Maximum	5
	Minimum	1
	Median	3
Chest infection		29 (16)
Post-traumatic acute respiratory distress syndrome		6 (3)
Sepsis		5 (3)
Ventriculitis		2 (1)

Categorial variables are represented as number (percentage) and continuous variables are represented as mean ± SD or median

### Diagnostic parameters

Straight sinus flow velocities, middle cerebral artery flow velocities, and optic nerve sheath diameter were correlated with intracranial pressure (*p* < 0.0001 for all). Arterial blood pressure (*p* = 0.127) and middle cerebral artery pulsatility index (*p* = 0.06) were not correlated with intracranial pressure (Table 2). Inter-rater reliability had strong agreement (intraclass correlation coefficient = 0.71) among ultra-sonographers.

**Table 2:** Diagnostic parameters

Characteristics		Value	r (Pearson correlation coefficient)	Significant range of ‘r’	*p*-value
Data of patients included in the analysis		185			
Intracranial pressure (mmHg)	Minimum	5	Reference	Reference	Reference
	Maximum	17			
	Mean ± SD	10.24 ± 3.51			
^a^Arterial blood pressure (mmHg)	Minimum	84	0.1125	0.2527–0.3233	0.127
	Maximum	101			
	Mean ± SD	90.61 ± 5.29			
Cerebral perfusion pressure (mmHg)	Minimum	71	0.9527	0.9243–0.957	< 0.0001
	Maximum	88			
	Mean ± SD	78.13 ± 4.58			
Partial pressure of carbon dioxide (mmHg)	Minimum	4.85	0.9027	0.872–0.9264	< 0.0001
	Maximum	5.95			
	Mean ± SD	5.24 ± 0.27			
Straight sinus systolic flow velocity (cm/s)	Minimum	23	0.8662	0.825–0.8982	< 0.0001
	Maximum	40			
	Mean ± SD	31.11 ± 4.9			
Straight sinus diastolic flow velocity (cm/s)	Minimum	12	0.5538	0.4451–0.6465	< 0.0001
	Maximum	18			
	Mean ± SD	13.47 ± 1.31			
Straight sinus means flow velocity (cm/s)	Minimum	15	0.9298	0.9072–0.947	< 0.0001
	Maximum	25			
	Mean ± SD	20.16 ± 3.23			
Middle cerebral artery systolic flow velocity (cm/s)	Minimum	95	0.8724	0.833–0.9031	< 0.0001
	Maximum	115			
	Mean ± SD	103.27 ± 5.64			
Middle cerebral artery diastolic flow velocity (cm/s)	Minimum	40	0.7681	0.7016–0.8214	< 0.0001
	Maximum	55			
	Mean ± SD	47.38 ± 4.26			
Middle cerebral artery means flow velocity (cm/s)	Minimum	60	0.9	0.8685–0.9243	< 0.0001
	Maximum	76			
	Mean ± SD	69.1 ± 3.81			
Optic nerve sheath diameter (mm)	Minimum	4	0.8717	0.832–0.9025	< 0.0001
	Maximum	6			
	Mean ± SD	4.77 ± 0.43			
^a^Middle cerebral artery pulsatility index	Minimum	0.85	0.1423	0.2134–0.2809	0.06
	Maximum	1.05			
	Mean ± SD	0.93 ± 0.08			

Variables are represented as mean ± SD

Pearson correlation was performed with intracranial pressure

A *p* value less than 0.05 was considered significant

^a^Insignificant correlation with intracranial pressure

### Prediction of mortality

From institutional records, it was found that 34 patients died during hospitalization and 13 patients died during the follow-up period. No significant association was observed between intracranial pressure and neuropsychological and functional outcomes during hospitalization and follow-up period for survived patients. Multivariate analysis was performed among diagnostic data of death patients. Intracranial pressure (*p* = 0.015) and optic nerve sheath diameter (*p* = 0.035) were found to be independent predictor of mortality (Table 3).

**Table 3:** Multivariate analysis for prediction of mortality

Characteristics	*p*-value
Data of patients included in the analysis	47
Intracranial pressure^a^	0.015
Arterial blood pressure	0.12
Cerebral perfusion pressure	0.072
Partial pressure of carbon dioxide	0.085
Straight sinus systolic flow velocity	0.062
Straight sinus diastolic flow velocity	0.067
Straight sinus means flow velocity	0.068
Middle cerebral artery systolic flow velocity	0.078
Middle cerebral artery diastolic flow velocity	0.086
Middle cerebral artery means flow velocity	0.082
Optic nerve sheath diameter^a^	0.035
Middle cerebral artery pulsatility index	0.095

A *p* value less than 0.05 was considered significant

Data of survived patients were considered as the reference standard

^a^Significant predictor of mortality

## Discussion

### Intracranial pressure measurement

In the study, all the enrolled patients required admission diagnosis and subjected to measure intracranial pressure. Also, elevated intracranial pressure was found a significant parameter of death (*p* = 0.015) and no association between intracranial pressure and neuropsychological outcome during follow-up period among survivors. The results of the study were consistent with prospective cohort studies [1, 5] and a multicenter, controlled trial [2]. The pathophysiological events and elevated intracranial pressure can have effects on neuropsychological outcome in patients with brain injury [16]. Intracranial pressure is an independent predictor of death in patients with brain injury.

### Venous transcranial Doppler measurements

Straight sinus flow velocities and middle cerebral artery flow velocities had a correlation with intracranial pressure, those were measured invasively but failed to predict mortality. The results of the study were consistent with the clinical study [2, 17] and with prospective observational study [5]. Increasing intracranial pressure leads to venous hemodynamic changes but strongly in the low-pressure venous compartment only [5]. Transcranial insonation difficulties and anatomical variations in cerebral veins were responsible for the poor performance of straight sinus and middle cerebral artery flow velocities to predict intracranial pressure [5]. Venous transcranial Doppler measurements are poorly developed method of diagnosis.

### Optic nerve sheath diameter measurements

Optic nerve sheath diameter was found an individual parameter of death (*p* = 0.035) as well as had correlation (*p* < 0.0001) with intracranial pressure, those were measured invasively. The results of the study were consistent with prospective observational studies [5, 7, 9, 18] and a multicenter, controlled trial [2]. The optic nerve is surrounded by cerebrospinal fluid [7]. If cerebrospinal fluid pressure increases, there will be enlargement of the optic nerve sheath [5]. Even, measurement of optic nerve sheath diameter is a simple, accurate, safe, and quick method of diagnosis. Clinicians would prefer the measurements of optic nerve sheath diameter in their intensive care units for patients with brain injury.

### Arterial transcranial doppler measurement

Middle cerebral artery pulsatility index had no correlation (*p* = 0.06) with intracranial pressure and was not independent parameter of death (*p* = 0.095). The results of the study were consistent with the clinical studies [5, 17] but not consistent with case report [19]. The possible justification is that in physiological parameters may affect pulsatility index [17] while intracranial pressure is independent parameter [20]. Even, intracranial pressure is a dynamic dimension that changes rapidly with time [7] and it is unclear whether or how pulsatility index would change. Pulsatility index is not a reliable predictor of intracranial pressure.

### Ultrasound versus invasive intracranial pressure measurement

During the study, besides the invasive intracranial pressure measurement method, optic nerve sheath diameter and arterial and venous transcranial Doppler measurements were performed for all patients who required measurements of intracranial pressure. Invasive intracranial pressure measurement devices are ‘gold standard’ modalities [21] but due to the lack of neurosurgeons and/or contraindications, it is not always feasible [5, 19]. Arterial and venous transcranial ultrasound is fast and does not require specific dedicated hardware [8]. Ultrasonography device is available in the intensive care unit too [5]. Ultrasonography equipment is a hands-free tool for patients who need to measure intracranial pressure.

### Limitations

Several limitations of the study have reported for examples, different types of brain injury patients were enrolled. Type of brain injury also has an effect on mortality. Effects of type of brain injury on mortality is not evaluated in multivariate analysis. There is a need for specialized training for image analysis of ultrasound. The threshold values for intracranial pressure and optic nerve sheath diameter are not well-defined for brain injuries [5]. Unlike an invasive method, ultra-sound has inter-and intra-observer variabilities.

## Conclusion

Non-invasively measured straight sinus flow velocities, middle cerebral artery flow velocities, and optic nerve sheath diameter were correlated with invasively measured intracranial pressure. Optic nerve sheath diameter and intracranial pressure were independent parameters for mortality. Ultrasound measurement can be successfully used instead of invasive intracranial pressure measurement method in patients with brain injury. The large human trial is recommended to validate non-invasive techniques.

## Data Availability

The datasets used and analyzed during the current study available from the corresponding author on reasonable request.

## Abbreviations

FVd: Diastolic flow velocity
FVm: Mean flow velocity
FVs: Systolic flow velocity
STROBE: Strengthening the reporting of observational studies in epidemiology
